# EEG Findings of Reduced Neural Synchronization during Visual Integration in Schizophrenia

**DOI:** 10.1371/journal.pone.0119849

**Published:** 2015-03-18

**Authors:** Jonathan K. Wynn, Brian J. Roach, Junghee Lee, William P. Horan, Judith M. Ford, Amy M. Jimenez, Michael F. Green

**Affiliations:** 1 Veterans Affairs Greater Los Angeles Healthcare System, Los Angeles, CA, United States of America; 2 Psychiatry and Biobehavioral Sciences, University of California Los Angeles, Los Angeles, CA, United States of America; 3 Veterans Affairs San Francisco Medical Center, San Francisco, CA, United States of America; 4 University of California San Francisco, San Francisco, CA, United States of America; Max Planck Institute for Human Cognitive and Brain Sciences, GERMANY

## Abstract

Schizophrenia patients exhibit well-documented visual processing deficits. One area of disruption is visual integration, the ability to form global objects from local elements. However, most studies of visual integration in schizophrenia have been conducted in the context of an active attention task, which may influence the findings. In this study we examined visual integration using electroencephalography (EEG) in a passive task to elucidate neural mechanisms associated with poor visual integration. Forty-six schizophrenia patients and 30 healthy controls had EEG recorded while passively viewing figures comprised of real, illusory, or no contours. We examined visual P100, N100, and P200 event-related potential (ERP) components, as well as neural synchronization in the gamma (30-60 Hz) band assessed by the EEG phase locking factor (PLF). The N100 was significantly larger to illusory vs. no contour, and illusory vs. real contour stimuli while the P200 was larger only to real vs. illusory stimuli; there were no significant interactions with group. Compared to controls, patients failed to show increased phase locking to illusory versus no contours between 40-60 Hz. Also, controls, but not patients, had larger PLF between 30-40 Hz when viewing real vs. illusory contours. Finally, the positive symptom factor of the BPRS was negatively correlated with PLF values between 40-60 Hz to illusory stimuli, and with PLF between 30-40 Hz to real contour stimuli. These results suggest that the pattern of results across visual processing conditions is similar in patients and controls. However, patients have deficits in neural synchronization in the gamma range during basic processing of illusory contours when attentional demand is limited.

## Introduction

Visual integration refers to the process by which basic local visual aspects, such as brightness, motion, and color, are combined to form a more complex visual percept, such as an actual object or shape [1]. Patients with schizophrenia have well-documented deficits in visual perception, including visual integration. The CNTRICS (Cognitive Neuroscience Treatment Research to Improve Cognition in Schizophrenia) Initiative prioritized visual integration as one of two key visual constructs for the study of schizophrenia [1]. Impairments in visual integration in schizophrenia are seen using various paradigms, including contour integration [2], perceptual closure [3], and facial processing [4]. Integration can occur through synchronization of cortical activity, as well as through feedback from higher neural levels to lower neural levels [5]. However, nearly all studies of visual integration have occurred in the context of an active attention task, requiring the participants to make some judgment on the object being presented. The current study was designed to examine in close detail the early perceptual processes involved with visual integration of illusory and real figures during a passive task.

**Fig 1:**
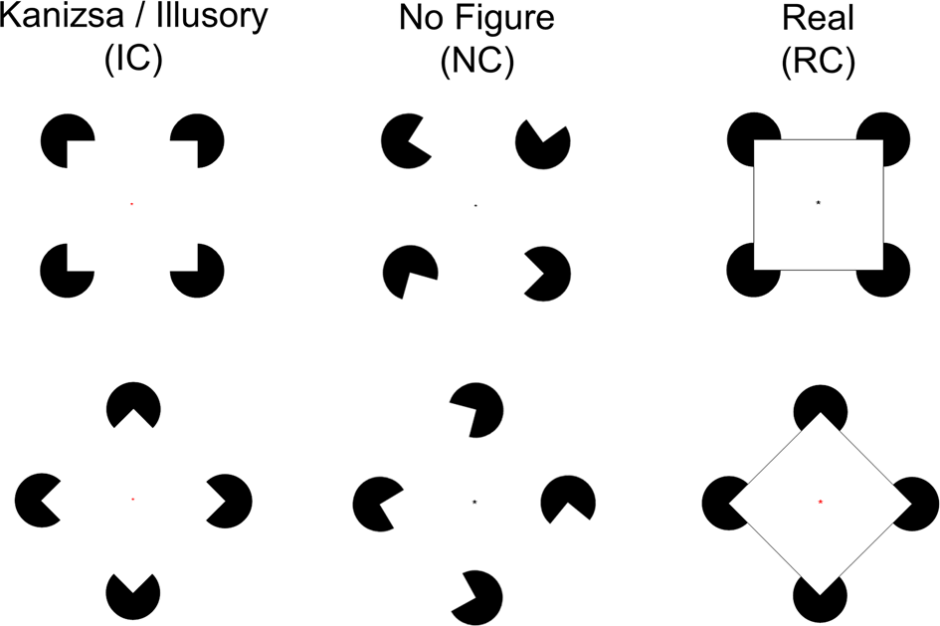
Example of stimuli, comprised either real figures (square or diamond) or no figures. Real figures were composed of either real contours (RC) or illusory contours (IC). No figure stimuli contained no contours (NC). A small fixation dot at the center of the stimulus changed from black to red and vice versa periodically.

In healthy samples, visual integration, in particular Gestalt processing, can be examined through the use of illusory contours (IC), known as Kanizsa figures [6] (see Fig. 1). The neural mechanisms of Kanizsa shape perception are well-described in non-clinical animal and human samples. Electroencephalography (EEG) and functional magnetic resonance imaging studies have shown that illusory contour processing is localized to bilateral lateral occipital cortex (LO) [7,8]. However, it has also been demonstrated that recurrent feedback information from LO to V1 and V2 [9] and even from V2 to V1 is also necessary for completion of illusory processing [10].

EEG studies in healthy populations using Kanizsa stimuli have shown that N100 event-related potentials (ERP) are larger to stimuli with IC versus those with no contours (NC) [11,12,13,14]. Similarly, EEG time-frequency analyses have shown increased gamma band (25–70) Hz neural synchrony (e.g., intertrial phase locking factor, PLF) to illusory vs. no contours [15]. Gamma-band synchronization is thought to be involved with feature-binding, a bottom-up process that “binds” distinct aspects of the same stimulus into a coherent object [16].

IC stimuli have been examined with EEG in schizophrenia using active tasks in which participants determine the presence or absence of an illusory figure. In these studies, patients exhibit an overall reduced P100 compared to controls, while both patients and controls exhibit a larger N100 to IC versus NC stimuli [15,17,18,19]. However, in one study with a passive task, patients showed intact P100 amplitudes, and neither group showed a larger N100 to IC versus NC stimuli [20]. Patients did exhibit a slightly prolonged P100 latency compared to healthy controls when viewing IC stimuli, indicating a delayed neural response when visual integration of illusory contours is necessary. Schizophrenia patients also have deficits in synchronizing activity within the gamma-band, between 30–60 Hz, during IC processing [15,17,18], suggesting that a failure to synchronize neural activity may underlie visual integration deficits. These deficits are potentially due to reported N-methyl-D-aspartate receptor hypofunction [21,22] located on inhibitory interneurons [23,24] which are thought to be responsible for EEG synchronization [25,26]. Moreover, gamma band deficits during illusory contour processing have been shown to be correlated with positive symptom severity (e.g. [15]).

The studies mentioned above that used IC stimuli in active tasks all yielded similar findings. However, it is not clear whether patients exhibit neural activation and gamma synchronization deficits when attention to the stimuli is limited, as only one previous study included such a task and found a different pattern of results. Hence, in the current study we had three aims. First, we examined differences in EEG measures of neural activity and synchronization to IC and NC stimuli that were matched for their physical characteristics. We hypothesized that healthy controls and schizophrenia patients would show larger N100 amplitude to IC versus NC figures, but only controls would show greater synchronization to IC stimuli. Second, we examined differences in neural activity and synchronization generated by IC and real contours (RC) (i.e., luminance-defined contours) in which the psychological experience was similar (i.e., impression of contour). We hypothesized that there would be a main effect of group (patients would show overall ERP amplitude and synchronization deficits compared to controls) but no effect of condition on neural activity or synchronization. Finally, we hypothesized that positive symptoms would be correlated with gamma band activity in the schizophrenia patients.

## Methods

### Participants

EEG data were initially collected in 48 patients with schizophrenia (40 male, 8 female) and 31 healthy controls (24 male, 7 female). Schizophrenia patients were recruited from outpatient treatment clinics at the Veterans Affairs Greater Los Angeles Healthcare System (GLA) and through presentations in the community. Forty-five patients were receiving second generation antipsychotic medication, 2 patients were receiving first generation antipsychotic medication, and 1 was taking both types of medication at the time of testing; medication information for 2 patients could not be obtained. All patients met diagnostic criteria for schizophrenia based on the Structured Clinical Interview for DSM-IV Axis I Disorders (SCID; [27]. Patients were between 18 and 60 years of age, and were excluded from participation if they had: substance abuse in the past month or dependence in the last six months, IQ < 70 based on examination of medical record, a history of loss of consciousness for more than one hour, an identifiable neurological disorder, or were not sufficiently fluent in English to consent and understand procedures. Psychiatric symptoms were evaluated using the expanded 24-item UCLA version of the Brief Psychiatric Rating Scale (BPRS; [28] and the Scale for the Assessment of Negative Symptoms (SANS) [29]. For the BPRS we report the total score and means for the “positive symptom,” “negative symptom,” “agitation/mania,” and “depression/anxiety” factors [30].

Healthy control participants (between 18–60 years of age) were recruited through advertisements in local newspapers and internet postings. An initial screening interview excluded potential healthy controls who had any identifiable neurological disorder or head injury, had a first-degree relative with schizophrenia or another psychotic disorder, were not sufficiently fluent in English, had a personal history of schizophrenia or other psychotic disorder, bipolar disorder, recurrent depression, had a lifetime history of substance dependence, or had any substance abuse in the last 6 months. Potential healthy control participants were interviewed with the SCID-I and portions of the Structured Clinical Interview for DSM-IV Axis II Disorders (SCID-II; [31], and were excluded if they had any of the following Axis II disorders: avoidant, borderline, paranoid, schizoid, or schizotypal.

All SCID interviewers were trained through the Treatment Unit of the Department of Veterans Affairs VISN 22 Mental Illness Research, Education, and Clinical Center (MIRECC) to a minimum kappa of 0.75 for key psychotic and mood items. All participants had the capacity to give informed consent and provided written informed consent after all procedures were fully explained in accordance with procedures approved by the Institutional Review Boards at the University of California, Los Angeles (UCLA) and GLA.

### Procedures

Participants viewed images (diamonds or squares) that contained illusory (IC), no (NC), or real contours (RC). Images were comprised of a series of inducers (“pacmen,” i.e. circle with a cut-out wedge) and/or lines that were arranged to form the figures (see Fig. 1). For the RC figures, lines were drawn between inducers to form a continuous contour; for IC figures, inducers were arranged to give the illusion that a figure was present; for NC figures, inducers were arranged such that no figure could be discerned.

Stimuli were presented on a 17-inch cathode ray tube computer monitor with a screen refresh rate of 160 Hz, positioned 1 m from the participant. Each inducer measured 1.55 degrees of visual angle, with a wedge that was created by removing 25% of the circle. One side of the real / illusory image (square or diamond) measured 3.72 degrees, resulting in a support ratio of 0.42 [32].

To ensure that participants were actively engaged with the task and were awake and watching the monitor, they were instructed to view a central fixation point that was present throughout the experiment and to push the mouse button whenever the color of the fixation changed from black to red and vice versa (33 times). Participants had 3000 ms to make a response. IC, RC and NC stimuli were displayed for 250 ms followed by an intertrial interval (ITI) that varied between 1700–3800 ms. Each of the six stimulus types (real, illusory, and no contour; square and diamond) were randomized in blocks of six trials for a total of 20 blocks, resulting in a pseudo-randomized fixed order of 120 trials. This same sequence was presented three times in a row, for a total of 360 trials. At most, two stimuli could be repeated in a row. The task took approximately 20 minutes to complete.

### EEG Recording and Processing

Participants had their EEG activity recorded continuously using a Neuroscan SynAmps^2^ amplifier (Compumedics USA, Charlotte, NC). Data were sampled at 500 Hz with filter settings of 1.0 to 100 Hz. Sixty-four equidistant cap-mounted, sintered Ag-AgCl electrodes were positioned using a modified international 10–20 system placement scheme. Additionally, four electrodes were used to measure horizontal electrooculogram (EOG; placed on the outer canthus of the left and right eye) and vertical EOG (placed above and below the left eye). All electrodes were referenced to a point halfway between electrodes Cz and CPz and a forehead ground was employed.

### EEG Analyses

#### ERP Waveforms

All ERP data were processed using BrainVision Analyzer 2.0 software (Brain Products, Germany). Data were re-referenced offline to the left and right mastoids. Eyeblinks were removed from the data using established mathematical procedures [33]. Data were epoched to 100 ms pre- and 600 ms post-stimulus onset and then low-pass filtered at 20 Hz (24 dB/Hz rolloff). Baseline correction on the 100 ms prior to stimulus presentation was applied. Artifact rejection was performed for any trial that exceeded +/−100 μV at any electrode site. Two patients and 1 control were excluded for having excessively noisy data due to continuous muscle artifact or electrode shorting; thus, the final sample size for analyses was 46 patients and 30 controls. For the remaining participants, there was no significant difference in the number of trials accepted for each figure type or for each group (see Table 1).

**Table 1:** Mean (SD) demographic information, symptom ratings, behavioral performance, and accepted EEG trials.

	Schizophrenia Patients (n = 46)	Healthy Controls (n = 30)
Age	45.8 (10.9)	42.4 (9.3)
Education*	12.6 (1.1)	14.1 (1.9)
Parental Education	13.3 (3.3)	14.7 (3.0)
Male:Female	40:6	23:7
**BPRS**		
Total Score	43.5 (10.8)	
Factors (mean score per item)		
Positive Symptoms	2.2 (0.9)	
Negative Symptoms	1.7 (0.8)	
Depression/Anxiety	1.8 (0.6)	
Agitation/Mania	1.4 (0.5)	
**Mean performance (out of 33)**	26.9 (7.9)	30.1 (6.2)
**Mean accepted EEG trials (out of 120)**		
Illusory Contours	100.7 (17.7)	104.4 (15.9)
No Contours	100.5 (18.1)	105.5 (15.1)
Real Contours	99.4 (18.3)	105.5 (15.7)

* p < 0.05 difference between groups.

**Fig 2:**
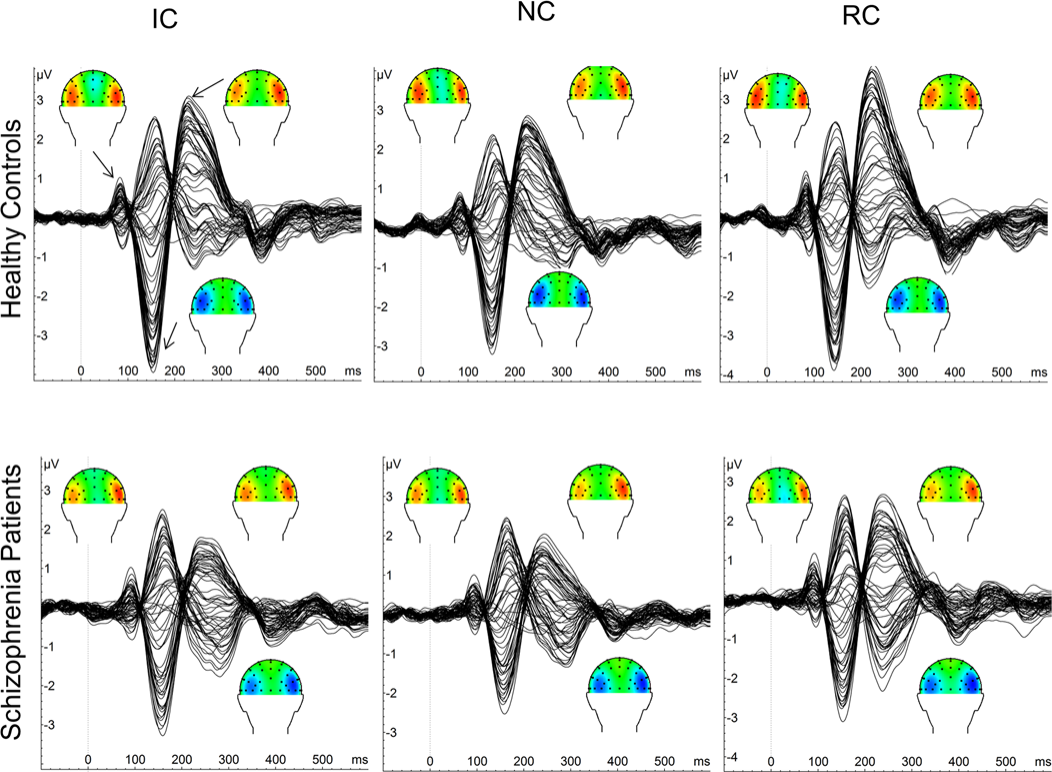
Butterfly plots for each group and figure type time locked to target onset (0 ms) for controls (top) and patients (bottom) for all three figure types (IC, NC, RC). Scalp current density (SCD) maps for the corresponding three main ERP components (P100, N100, P200) are plotted.

We examined three separate ERP components: the P100, N100 and P200. A 40 ms time window of activity (+/- 20 ms around the peak) was defined for each ERP component based on the peak activity observed by inspection of butterfly plots and scalp current density (SCD) maps (see Fig. 2). SCD maps are reference-free and reduce the effect of volume conduction due to current flow within the scalp, thus approximating the source of intracranial generators and help narrow down the number of electrodes to analyze [14]. Based on inspection of the SCD maps where activity was most prominent for each component, we examined activity averaged across electrodes P3, P5, P7, PO3, PO5, and PO7 (left hemisphere) and P4, P6, P8, PO4, PO6, PO8 (right hemisphere). The mean EEG activity within each window was used for the main dependent measure. The time windows for each component and each task for both groups are shown in Table 2. We additionally examined P100 latency in order to compare our results to those of Ikeda et al. [20].

**Table 2:** Time-windows (in ms) of analysis for schizophrenia patients and healthy controls for illusory, no contour, and real figures.

P100	Patients	Controls
Illusory Contour	70–110	66–106
No Contour	72–112	64–104
Real Contour	70–110	64–104
**N100**		
Illusory Contour	136–176	134–174
No Contour	138–178	134–174
Real Contour	134–174	128–168
**P200**		
Illusory Contour	230–270	222–262
No Contour	232–272	224–264
Real Contour	220–260	210–250

#### Time Frequency Analysis

After eyeblink correction, data were epoched to 1000 ms pre- and 1500 ms post-stimulus onset. No filtering was applied. We examined activity for the same electrodes used in the ERP analysis. Artifact rejection was performed on these electrodes between 250 ms pre- and 750 ms post-stimulus, so that any trial with activity within this window that exceeded +/−100 μV was rejected.

Time-frequency analysis was performed using the FieldTrip plugin for Matlab [34]. A Morlet wavelet decomposition was performed across all time points and all frequencies between 2–70 Hz. The wavelet’s Gaussian shape was defined by a constant ratio (σ_f_ = f/7) and wavelet duration (3σ_t_), where f is the center frequency and σ_t_ = 1/(2πσ_f_). At 40 Hz, the wavelet duration is over six cycles (4σ_t_ = 167.1 ms) with a spectral bandwidth of 4σf = 34.2857 Hz. FieldTrip performed wavelet decompositions by multiplying the Fast Fourier Transform (FFT) of the wavelet by the FFT of the signal. The inverse FFT of the resultant is adjusted so that the time course of the data corresponds to the time course of the original signal. These calculations are conducted in 1 Hz steps. Each trial was analyzed with this method after which the PLF was determined (calculated as 1 minus the phase variance). Larger PLF values at a specific frequency within a specific time window indicate that oscillations have become phase-synchronized across trials with respect to event onset. Based on visual inspection of the time frequency plots, we identified two separate bursts of activity between 70–100 ms: one between 30–40 Hz and the other between 40–60 Hz. We therefore examined mean PLF between 70–100 ms separately for these two gamma bursts, averaged across all electrodes examined. This time-frequency window was similar to time-frequency windows where early gamma band responses have been reported [13,15,18].

#### Statistical Analysis and Key Contrasts

We conducted two sets of analyses each for the ERP and PLF data to address our hypotheses. The first set of analyses focused on the contrast between IC and NC stimuli because these are matched for the physical characteristics of the stimuli on the screen. The second focused on the contrast between IC and RC stimuli, in which the psychological experience is similar. Repeated measures analysis of variance (ANOVAs) were used with figure type and hemisphere as the within subject factor, and group was the between subject factor. We report effect sizes as partial eta-squared (ƞ_p_^2^). Follow-up Bonferonni-corrected t-tests were used to examine significant main effects or interactions. An alpha level of p = 0.05 was used for all ERP and PLF analyses. Finally, we examined correlations between the positive BPRS factor and its associated items and ERP and PLF measures to all three stimulus types (IC, RC and NC).

## Results

### Demographic and Clinical Characteristics

Table 1 lists the group demographics, symptom ratings on the BPRS for the patients, mean behavioral performance, and mean number of accepted EEG trials per figure type for the final sample of 46 patients and 30 controls. As most of our patient participants were recruited from VA clinics the sample is predominantly male. Patients were clinically stable and exhibited mild clinical symptoms. For EEG, there was no significant difference in mean (SD) performance in detecting the color change between patients and controls.

### Illusory vs. No Contour Analyses

#### ERP Results

**Fig 3:**
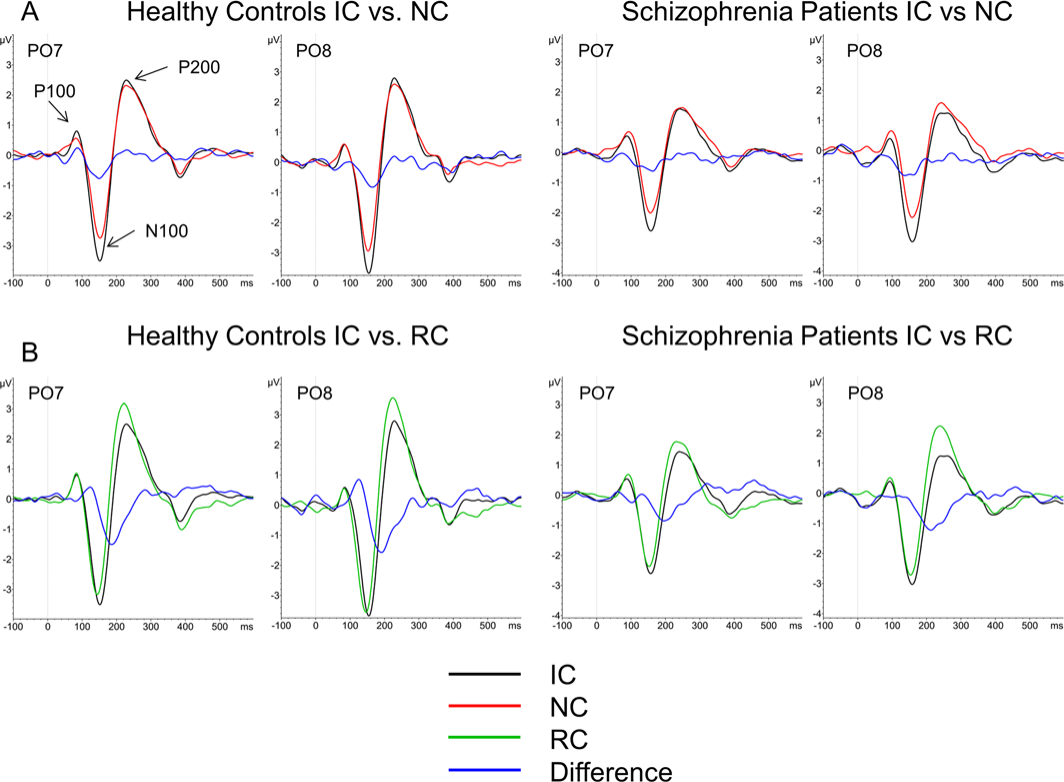
ERPs at electrodes PO7 and PO8, with the P100, N100 and P200 marked with arrows. A) ERPs comparing the IC (black) and NC (red) stimuli along with the IC-NC difference (blue). B) ERPs comparing the IC (black) and RC (green) stimuli along with the IC-RC difference (blue).

Representative waveforms for electrodes PO7 and PO8 are shown in Fig. 3A. Waveforms for IC (black) and NC (red) and the difference between the respective conditions (blue), are plotted. The P100, N100, and P200 responses are marked with arrows. Mean amplitudes for each ERP for each group are shown in Table 3.

**Table 3:** Mean (SD) ERP amplitudes and phase locking factor (PLF) values for patients and controls for illusory, no contour, and real figures.

	Patients		Controls	
P100	Left	Right	Left	Right
Illusory Contour	0.37 (0.96)	0.41 (1.06)	0.56 (0.66)	0.39 (0.75)
No Contour	0.53 (0.82)	0.53 (0.93)	0.52 (0.65)	0.45 (0.73)
Real Contour	0.54 (0.92)	0.41(0.99)	0.59 (0.73)	0.31 (0.93)
**N100**				
Illusory Contour	-2.15 (1.74)	-2.63 (2.12)	-3.07 (2.08)	-3.21 (2.06)
No Contour	-1.61 (1.44)	-1.92 (1.64)	-2.41 (1.77)	-2.47 (1.89)
Real Contour	-1.95 (1.46)	-2.37 (1.86)	-2.86 (2.15)	-3.06 (2.10)
**P200**				
Illusory Contour	1.41 (1.37)	1.51 (1.61)	2.43 (1.65)	2.64 (1.76)
No Contour	1.44 (1.31)	1.67 (1.70)	2.34 (1.54)	2.42 (1.76)
Real Contour	1.81 (1.74)	2.31 (2.19)	3.15 (1.79)	3.39 (2.01)
**PLF: 30–40 Hz**				
Illusory Contour	0.11 (0.04)	0.11 (0.04)	0.12 (0.07)	0.14 (0.09)
No Contour	0.10 (0.04)	0.10 (0.04)	0.13 (0.08)	0.14 (0.10)
Real Contour	0.11 (0.05)	0.12 (0.06)	0.17 (0.08)	0.18 (0.09)
**PLF: 40–60 Hz**				
Illusory Contour	0.10 (0.03)	0.10 (0.03)	0.13 (0.07)	0.13 (0.07)
No Contour	0.09 (0.02)	0.10 (0.02)	0.11 (0.05)	0.11 (0.05)
Real Contour	0.10 (0.03)	0.11 (0.03)	0.12 (0.08)	0.13 (0.09)

#### P100

There were no significant main effects of group, figure type or hemisphere, and no significant interactions for amplitude. There was a significant effect of hemisphere for latency, (F_1, 74_ = 4.19, p < 0.05, ƞ_p_^2^ = 0.05), with faster latencies in the left vs. right hemisphere, 83.2 (12.1) and 85.8 (11.8) ms, respectively.

#### N100

There was a marginally significant main effect of group (F_1, 74_ = 3.22, p < 0.08, ƞ_p_^2^ = 0.04), a significant main effect of figure type (F_1, 74_ = 34.74, p < 0.001, ƞ_p_^2^ = 0.32), and a significant main effect of hemisphere (F_1, 74_ = 4.72, p < 0.05, ƞ_p_^2^ = 0.06). There were no significant interactions. The marginal effect of group was because controls tended to have a larger N100 than patients, -2.85 (1.91) μV vs. -2.10 (1.53) μV, respectively. The main effect of figure type was due to larger responses to IC vs. NC figures, -2.69 (1.93) and -2.03 (1.61) μV, respectively. The main effect of hemisphere was due to larger responses in the right vs. left hemisphere, -2.50 (1.87) vs. -2.22 (1.70) μV, respectively.

#### P200

There was only a significant main effect of group (F_1, 74_ = 7.88, p < 0.01, ƞ_p_^2^ = 0.10), due to controls having a significantly larger P200 than patients, 2.46 (1.56) μV vs. 1.51 (1.37) μV, respectively (see Table 3 for means).

#### Phase Locking Factor Results

**Fig 4:**
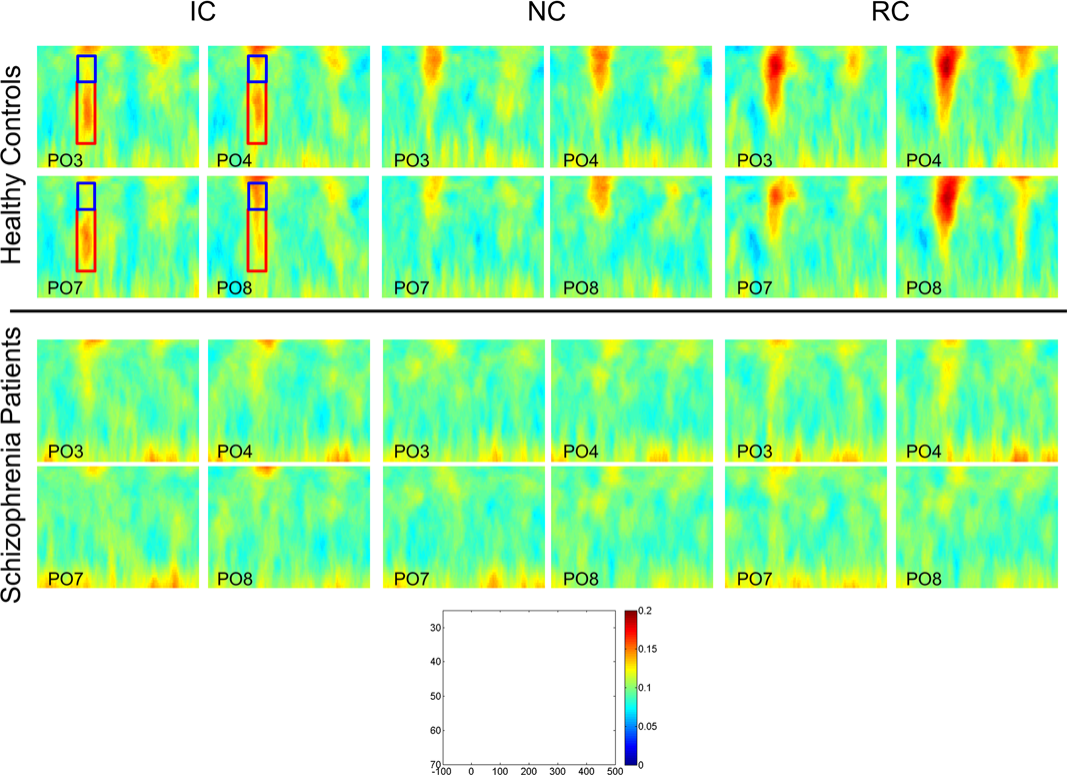
Time-frequency plots of phase locking factor for controls (top) and patients (bottom) for all three figure types. Plots from four representative electrodes (PO3, PO4, PO7, PO8) are shown. The scale for each plot is included. Frequency is plotted on the y-axis and ranges from 25–70 Hz (from top to bottom); time from onset of the stimulus is plotted on the x-axis and ranges from-100 to 500 ms. The blue box highlights the 30–40 Hz band and the red box the 40–60 Hz band that were analyzed.

Time-frequency plots of phase locking at representative electrodes PO3, PO4, PO7, and PO8 are shown in Fig. 4. We examined mean PLF (across all electrodes) separately within the 30–40 Hz and 40–60 Hz frequency band between 70–100 ms, and these values are shown in Table 3. For the 30–40 Hz band, there was a significant main effect of group, F_1, 74_ = 4.15, p < 0.05, ƞ_p_^2^ = 0.05. Patients had significantly lower PLF compared to controls, 0.11 (0.02) vs. 0.13 (0.07), respectively. The main effect of figure type and the figure type x group interaction were not significant.

For the 40–60 Hz band, there was a significant main effect of group, F_1, 74_ = 5.73, p < 0.02, ƞ_p_^2^ = 0.07, a significant main effect of figure type, F_1, 74_ = 9.56, p < 0.005, ƞ_p_^2^ = 0.11, and a significant group x figure type interaction, F_1, 74_ = 5.34, p < 0.03, ƞ_p_^2^ = 0.07. The interaction was due to controls having significantly greater PLF to IC compared to patients, 0.13 (0.07) and 0.10 (0.02), respectively, t_74_ = 2.91, p < 0.01. There was no significant difference between controls and patients for NC, 0.11 (0.05) and 0.10 (0.02), respectively, p < 0.24.

### Illusory vs. Real Contour Analyses

#### ERP Results

Representative waveforms for electrodes PO7 and PO8 are shown in Fig. 3B. Waveforms for IC (black) and RC (red) and the difference between the respective conditions (blue), are plotted. Mean amplitudes for each ERP for each group are shown in Table 3.

#### P100

There were no significant main effects or interactions for amplitude. For latency there was a significant group x figure type interaction, (F_1, 74_ = 6.37, p < 0.02, ƞ_p_^2^ = 0.08). Healthy controls had significantly faster latencies to RC vs. IC stimuli, 81.4 (11.0) and 85.2 (9.6) ms, respectively, t_29_ = 2.55, p < 0.02. There was no significant latency difference in patients for RC vs. IC stimuli, 86.5 (11.5) and 85.0 (12.4) ms, respectively.

#### N100

There was a marginally significant main effect of group (F_1, 74_ = 3.38, p < 0.07, ƞ_p_^2^ = 0.04), a significant main effect of figure type (F_1, 74_ = 4.41, p < 0.05, ƞ_p_^2^ = 0.06), and a significant main effect of hemisphere (F_1, 74_ = 6.77, p < 0.02, ƞ_p_^2^ = 0.08). There were no significant interactions. The marginal effect of group was because controls tended to have a larger N100 than patients, -3.05 (2.01) μV vs. -2.27 (1.66) μV, respectively. The main effect of figure type was due to larger responses to IC vs. RC figures, -2.69 (1.93) and -2.48 (1.82) μV, respectively. The main effect of hemisphere was due to larger responses in the right vs. left hemisphere, -2.75 (1.98) vs. -2.41 (1.82) μV, respectively.

#### P200

There was a significant main effect of group (F_1, 74_ = 9.12, p < 0.01, ƞ_p_^2^ = 0.11), a significant main effect of figure type (F_1, 74_ = 34.76, p < 0.001, ƞ_p_^2^ = 0.32), a significant main effect of hemisphere (F_1, 74_ = 5.25, p < 0.02, ƞ_p_^2^ = 0.07), and a significant figure type x hemisphere interaction (F_1, 74_ = 3.98, p < 0.05, ƞ_p_^2^ = 0.05). The main effect of group was because controls had a larger response than patients, 2.91 (1.65) μV vs. 1.76 (1.60) μV, respectively. The main effect of figure-type was due to larger responses to RC than IC stimuli. The figure type x hemisphere interaction was due to larger responses to RC stimuli in the right vs. left hemisphere, 2.74 (2.17) μV vs. 2.34 (1.87) μV, respectively; there was no difference between hemispheres to IC stimuli.

#### Phase Locking Factor Results

Time-frequency plots of phase locking at representative electrodes PO3, PO4, PO7, and PO8, are shown in Fig. 4. For the 30–40 Hz band, there were significant main effects of figure type, F_1, 74_ = 9.91, p < 0.005, group, F_1, 74_ = 8.41, p < 0.005, and a significant figure type x group interaction, F_1, 74_ = 6.48, p < 0.02. The significant interaction was due to controls having significantly greater PLF to RC than IC stimuli, 0.17 (0.09) and 0.13 (0.07), respectively, t _29_ = 3.21, p < 0.005. There was no significant difference between RC and IC stimuli for the patients, 0.11 (0.04) vs. 0.11 (0.03), respectively.

For the 40–60 Hz band, there was only a significant main effect of group, F_1, 74_ = 6.18, p < 0.02, ƞ_p_^2^ = 0.08, due to controls having a larger PLF compared to patients, 0.13 (0.07) vs. 0.10 (0.02), respectively.

### Correlations between Symptom Ratings and EEG Measures

We first correlated the scores on the BPRS positive factor with each of the EEG measures (ERP and PLF). A significant negative correlation between the scores on BPRS positive factor and PLF values between 40–60 Hz in the right hemisphere to the IC figures, r = -0.34, p < 0.03 was found. In particular, the unusual thought content (r = -0.43, p < 0.001) and bizarre behavior (r = -0.30, p < 0.05) items accounted for this finding. PLF values between 30–40 Hz in the right hemisphere to RC figures also correlated on BPRS positive factor, r = -0.34, p < 0.03, in particular with unusual thought content, r = -0.44, p < 0.01. As an exploratory analysis, we examined additional correlations with the BPRS (depression/anxiety, agitation/mania, total) and the SANS global scores. PLF values between 30–40 Hz in the right hemisphere to RC figures correlated with total anhedonia scores on the SANS, r = 0.35, p < 0.02. There were also significant correlations between the scores on the BPRS agitation/mania factor and P100 amplitude in the left (0.41, p < 0.01) and right hemisphere (0.36, p < 0.02) to IC figures. Given the large number of these additional correlations performed they would most likely not survive family-wise error correction and should be interpreted with caution.

## Discussion

This study examined the neural mechanisms involved with dysfunctional visual integration in schizophrenia while limiting the influence of attentional processing using EEG methodologies. Several key findings emerged from this study. First, patients and controls showed a similar pattern of ERP responses to the different figure types: both groups showed greater N100 amplitudes for IC compared to NC and both groups showed greater P200 amplitudes for RC compared to IC. Second, controls but not patients exhibited larger phase locking to IC compared to NC. Third, the positive BPRS factor (and items of unusual thought content and bizarre behavior) correlated with PLF values to IC stimuli in the right hemisphere. The results of this study demonstrate dysfunctional neural synchronization associated with visual integration in schizophrenia, even in the absence of specific attentional demands.

Regarding the key contrast between IC and NC stimuli, we found that the pattern of ERPs for illusory contour processing were intact in schizophrenia patients, consistent with previous ERP [11,12,14] and behavioral [35] findings. We found a non-significant trend for patients to show a lower N100 than controls, similar to other findings in the literature [15,17,18,19]. A novel finding of the current study is that, unlike controls, schizophrenia patients failed to show phase locking in the early (<100 ms) 40–60 Hz gamma band response to IC stimuli, suggesting poor neural synchronization during sensory processing may underlie visual integration deficits. Our findings are consistent with findings from other studies suggesting integration deficits can occur at very early stages of visual processing in V1/V2 [36]. Moreover, PLF to IC stimuli was correlated with positive symptoms of the BPRS, similar to prior studies [15,17,18]. As area LO has been implicated in the processing of real and illusory contours, the results of the current study suggest a deficit of neural synchronization within this area in patients with schizophrenia.

Regarding the contrast between IC and RC, patients had a trend for lower N100 amplitudes, both groups had larger P200 amplitudes to RC stimuli, and patients had overall reduced PLF across conditions. Little is known about the nature of the visual P200 ERP component or its role in schizophrenia. A previous study in healthy controls suggested that the P200 was involved with implicit categorization of stimuli [37]. The finding of increased P200 to RC stimuli could be consistent with this interpretation, given that a figure of real contours is easily categorized as a shape, whereas IC may be less implicitly categorized.

The finding of a correlation between PLF in the gamma band to IC stimuli and positive symptoms is consistent with prior behavioral and EEG studies. A recent behavioral study in schizophrenia found that patients characterized as disorganized were less capable than non-disorganized patients and healthy controls at discriminating visually completed shapes, but were not impaired at forming illusory contours [35]. Prior EEG studies have shown correlations between gamma band activity to illusory stimuli and positive symptoms as well [15,17,18]. While a tentative hypothesis, it is thought that the ability to organize thoughts and behavior shares the same neural process as visual integration [35]. It may be possible that when one process is dysfunctional it leads to dysfunction in the other.

One key difference between the current study and other EEG studies of visual integration in schizophrenia is that we utilized a task that attempted to limit attentional demand in processing the stimuli. It has been shown that the visual P100 and N100 [38] as well as activity in the gamma band [39] is larger in active vs. passive tasks, reflecting the influence of top-down attentional effects on visual processing. This would suggest that early gamma band abnormalities may originate at a higher level of processing [15,40]. However, it is interesting that gamma band abnormalities in patients remained even when attentional demand was limited in the current study, suggesting that gamma synchronization deficits in patients occurs even in the absence of top-down attentional effects and may originate at a lower level of processing. The lack of a P100 deficit in patients in the current study, in contrast to prior studies [15,17,18,19], is likely due to the nature of the task. Given these mixed effects of attention on the current findings, it would have been beneficial to have an active attention task as a comparison.

Our amplitude, but not latency, results are largely consistent with those of Ikeda et al. [20], where there was no group differences reported for P100 or N100 (referred to as “N200” in their study) amplitudes. In contrast to our results, though, Ikeda reported an effect of stimulus type for the P100 amplitude, with amplitudes being largest for real contours, smallest for no contours, and intermediate for illusory contours. In our latency results, however, we did not find slower P100 latencies to IC vs. RC stimuli in patients; rather, we found this pattern in healthy controls, though the difference (+3.8 ms) was very small and likely not very meaningful. A number of differences between that study and the current study (e.g., electrode location, number of subjects, size of stimuli, global field power for calculation of peak latency) could have potentially led to the differences in our results and those of Ikeda et al.

We also noticed a group by condition interaction for the RC and IC stimuli between 30–40 Hz that resulted from significantly greater PLF to RC vs. IC stimuli in healthy controls but not patients. It is not entirely clear why RC stimuli generated greater gamma phase-locking than IC stimuli. One possibility for this finding could be that RC stimuli automatically captured attention in controls, despite efforts to limit the role of attention to the stimuli. The enhanced PLF to RC vs. IC stimuli also coincided with a small, but significant, faster latency to RC stimuli in controls, also consistent with attention effects. As mentioned previously, attention to stimuli increases gamma activity as well as decreases ERP latencies. This possibility will require replication in future studies.

We found that patients had synchronization deficits in the gamma band when viewing illusory contours arising fairly early (< 100 ms), consistent with findings of previous studies [18]. However, other studies have suggested that illusory contour formation occurs between 100–200 ms after stimulus onset [7,9,19,41]. The later time periods have been linked more to the conscious perception of an illusory contour, whereas the earlier time period is linked more to the processing of physical properties of the stimulus. It may be the case that the observed deficits in schizophrenia during early visual processing are due to problems in the initial stage of processing of physical properties of the stimulus that lead to subsequent disruptions in visual integration, though that possibility remains speculative.

This study had a few limitations. First, patients were medicated at the time of testing and this could have potentially affected the results. However, it is unlikely antipsychotic medication could have affected the results, as previous studies have found no adverse effects on ERPs [42,43] and second generation medications have been shown to enhance gamma band responses [44]. Second, there is a possibility that the groups differ in their rate of microsaccades and that may have affected the time-frequency analysis [45]. However, this explanation is unlikely for two reasons: First, the effects of microsaccades are noted only for induced activity occurring approximately 200–300 ms after stimulus onset, and our effects on gamma were earlier; second, the observed increase in gamma in controls for IC is not consistent with the observation that there are generally fewer microsaccades to IC vs. NC contour stimuli [46].

Visual processing deficits are core features of schizophrenia and contribute to poor functioning. The results of the current study provide further information about the neural substrates of one key aspect of visual processing, namely visual integration. We found that visual integration is disrupted at an early visual processing stage, even when attentional demands are limited. While certain aspects of visual integration may be intact in schizophrenia, such as the increased N100 responses to IC stimuli, failures to synchronize activity within the gamma band may lead to further processing deficits.

## Supporting Information

S1 Data SPSS file containing all data presented in the results. (SAV)
